# Structural, Optical and Electrical Properties of Al+MoO_3_ and Au+MoO_3_ Thin Films Prepared by Magnetron Codeposition

**DOI:** 10.3390/ma14040766

**Published:** 2021-02-06

**Authors:** Tihomir Car, Ivan Jakovac, Ivana Šarić, Sigrid Bernstorff, Maja Micetic

**Affiliations:** 1 Ruđer Bošković Institute, Bijenička Cesta 54, 10000 Zagreb, Croatia; saric.ivana@irb.hr (I.Š.); maja.micetic@irb.hr (M.M.); 2 Physics Department, University of Zagreb, Bijenička Cesta 46, 10000 Zagreb, Croatia; ijakovac@phy.hr; 3 Elettra-Sincrotrone S.C.p.A., Strada Statale 14 km 163.5 in AREA Science Park, Basovizza, 34149 Trieste, Italy; sigrid.bernstorff@elettra.trieste.it

**Keywords:** gold, aluminum, molybdenum trioxide, nanoparticles, GISAXS, electrical resistivity

## Abstract

Structural, optical and electrical properties of Al+MoO_3_ and Au+MoO_3_ thin films prepared by simultaneous magnetron sputtering deposition were investigated. The influence of MoO_3_ sputtering power on the Al and Au nanoparticle formation and spatial distribution was explored. We demonstrated the formation of spatially arranged Au nanoparticles in the MoO_3_ matrix, while Al incorporates in the MoO_3_ matrix without nanoparticle formation. The dependence of the Au nanoparticle size and arrangement on the MoO_3_ sputtering power was established. The Al-based films show a decrease of overall absorption with an Al content increase, while the Au-based films have the opposite trend. The transport properties of the investigated films also are completely different. The resistivity of the Al-based films increases with the Al content, while it decreases with the Au content increase. The reason is a different transport mechanism that occurs in the films due to their different structural properties. The choice of the incorporated material (Al or Au) and its volume percentage in the MoO_3_ matrix enables the design of materials with desirable optical and electrical characteristics for a variety of applications.

## 1. Introduction

Molybdenum trioxide (MoO_3_) is an important transition metal oxide that has attracted the attention of researchers. Pure MoO_3_ shows a lot of fascinating optical and electrical properties, which have led to notable technological applications [1,2,3]. As a result of oxygen vacancies, MoO_3_ is also a wide n-type semiconductor with high ionic conductivity [4].

The technological applicability of novel materials largely depends on their nanostructural properties [2,5,6,7]. It is shown that the properties of the material are significantly influenced by the metal atoms, or nanostructures, “inserted” into the basic matrix [8,9,10,11]. In this context, the MoO_3_ matrix shows significant potential for improving its basic electrical and optical properties [10,11,12,13,14].

The dimensionality of functional materials, for example one, two and three dimensional, has been demonstrated as a dominant factor in determining the performance of the resulting applications [15]. Zero dimensional quantum dots (QDs) [16,17], one dimensional nanofibers/nanotubes/nanorods [18], two dimensional layered semiconductors/insulators [19] and three dimensional complex structures [20] have recently shown the most attractive features in terms of structural, optical, electrical and mechanical characteristics, thus creating the conditions for significant technological breakthroughs.

An important part of the development and research of new functional materials is related to the methods of preparation and conditioning of nanostructures in matrices [21,22]. The creation of spatially ordered nanoparticle (NP) lattices in the matrix material is not a necessary consequence of the magnetron deposition. Creation and self-organization of NPs are the result of physical processes of surface diffusion and aggregation of materials during deposition and also as interactions of neighboring clusters [23]. The creation mechanism of spatially arranged Ge and Ge/Si NPs in an alumina matrix has already been well researched and experimentally confirmed in our previous work [24,25,26].

Due to their chemical inertness, ease of preparation and interesting electronic, optical and medical properties, Au NPs have recently become the subject of increasing research. The potential use of Au NPs in cancer treatment [27,28] (photo-thermal effects, drug carriers), diagnostics (contrast agents), sensors and in chemical catalysis has been investigated [29]. An important property of Au NPs is their interaction with electromagnetic radiation by surface plasmon resonance [30,31]. Al is another very interesting material for addition in wide bandgap matrices because it can significantly influence their optical and electrical properties [32].

Al is also a cheap material compared with Au, and it also has interesting properties and applications. Although MoO_3_, Au and Al are well investigated as separate materials, there are not many investigations into their combination. Au+MoO_3_ films have been prepared, and the material shows advanced properties for application in solar cells [33], while the same combination with Al is very rarely investigated.

In this study, we investigated the structural, optical and electrical properties of Al+MoO_3_ and Au+MoO_3_ thin films prepared by magnetron sputtering codeposition. We demonstrate very different properties of the Al- and Au-based films. The Al atoms seem to incorporate in the MoO_3_ lattice without forming NPs. On the other hand, Au forms NPs that are regularly distributed within the MoO_3_ matrix. The overall absorption decreases with increasing Al content in the Al-based films, while it increases in the Au-based ones. Additionally, surface plasmon resonance is observed in the Au-based film with an NP-size-dependent peak position. Finally, the electrical resistivity of the films increases with the Al content, while it decreases with the Au content in the films due to different transport mechanisms.

## 2. Materials and Methods

Thin Au+MoO_3_ and Al+MoO_3_ films were prepared by magnetron simultaneous deposition in a multi-source sputtering system (CMS-18 from K.J. Lesker company, Glassport, PA, USA). All samples were deposited onto glass (VitroGnost microscope slides) and Si substrates at room temperature (300 K). The working gas pressure was p(Ar) = 0.47 Pa. Circular targets, 7.62 cm in diameter, were used. For each metal, three different volume ratios of metal (Al and Au) to MoO_3_ matrix were explored. The metal DC sputtering power density was kept constant (P_Au_ = 0.13 and P_Al_ = 0.22 W/cm^2^), while the RF deposition power density of the ceramic oxide MoO_3_ matrix was varied (1.64, 2.19 and 3.29 W/cm^2^). Thus, volume of metal to matrix ratios of 0.10, 0.12 and 0.20 were obtained. These volume ratios were determined from the deposition speed of each element. All films were deposited with a duration of 30 min.

The films are named after the used metal followed by the number showing its amount in the films. Thus, the film with the lowest Au amount is Au1, while the highest amount is Au3. The deposition conditions together with the sample names and film thicknesses and Au/Al to MoO_3_ volume ratios are summarized in Table 1.

Structural analysis of the films (the nanoparticle formation, their size and arrangement properties) was performed by grazing-incidence small-angle X-ray scattering (GISAXS). Grazing-incidence wide angle X-ray scattering (GIWAXS) was applied to determine the crystalline structure of the materials. Both measurement types were performed simultaneously at the Austrian SAXS beamline of Elettra-Sincrotrone in Trieste, using 8 keV photons and a 2D 100k Pilatus (for GIWAXS) and a 2D Pilatus3 1M (for GISAXS) detector system (Dectris Ltd., Baden, Switzerland). The GISAXS and GIWAXS maps were measured using grazing-incidence angles slightly above the critical angle.

Optical measurements were carried out using Ocean Optics (Orlando, FL, USA) equipment including a deuterium–halogen light source (DH-2000-BAL), a UV/VIS detector (HR4000) and SpectraSuite software.

The transport properties of the films were investigated by the measurement of the surface resistance using the van der Pauw four contact method [34] at room temperature. Indium contacts were placed on the sample edges. All current–voltage (I-V) measurements were done with a Keithley 2401 Sourcemeter SMU, controlled by a LabView program through which data were also collected.

## 3. Results

### 3.1. Structural Properties of the Films

#### 3.1.1. Nanoparticle Formation and Size—Arrangement Properties

**Figure 1:**
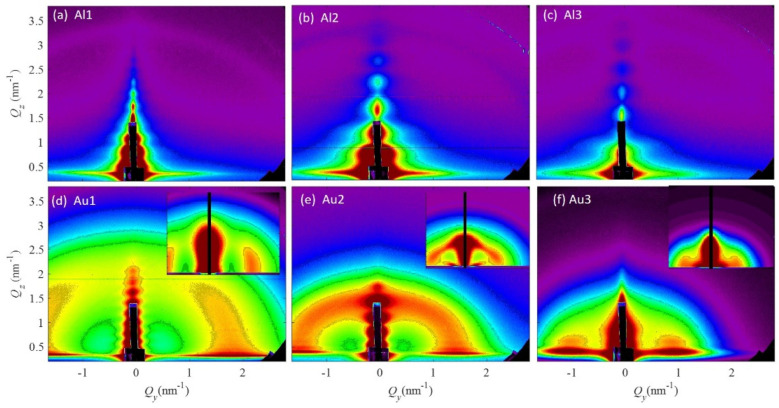
GISAXS maps of the investigated films. (**a**–**c**) Al-based films, (**d**–**f**) Au-based films. The insets in (**d**–**f**) show simulations of the measured GISAXS maps obtained by their numerical analysis.

The nanoparticle formation and structural properties were analyzed using the GISAXS technique. This method is very suitable for the analysis of nanostructured thin films as it provides structural parameters with excellent statistics. GISAXS maps of the investigated films are shown in Figure 1.

The GISAXS maps of the Al-based films are shown in Figure 1a–c and show no signal related to NP formation. The features in the center of their GISAXS maps originate from the coherent scattering, surface roughness and the entire film thickness contributions. These features are not interesting for further analysis of the NP structural and arrangement properties, as the NPs are not formed in the Al-based films according to the GISAXS analysis. It seems that all atoms are homogeneously distributed through the films.

However, the Au-based films, shown in Figure 1d–f, all have a characteristic semicircular (ring-like) signal, which shows the presence of NPs that have a correlated mutual first neighbor spacing (inter-nanoparticle distance). The same type of signal that is present in the Al-based films is present also in the maps of the Au-based films, but as we mentioned before is not interesting for further analysis. The radius of the ring-like signal in the Au-based films decreases with the fraction of Au in the films (Au1–Au3), showing an increase in the distance between the NPs and in the NP size. For the details of the structural properties, we performed a numerical analysis of the GISAXS maps.

The insets show simulations of the measured GISAXS maps obtained by numerical analysis of the GISAXS maps. For the numerical analysis we used the model described in Reference [35]. More precisely, we assumed that NPs order in a 3D paracrystal lattice, described by basis vectors ***a***_1_–***a***_3_. The vectors ***a***_1_ and ***a***_2_ are placed in the plane parallel to the substrate surface, while ***a***_3_ describes the ordering of the NPs in the direction perpendicular to the film surface. We assumed the ordering of the NPs in a body centered tetragonal lattice. A short range ordering is assumed along all basis vectors. The disorder in the NP lattice, i.e., the deviations from the ideal positions defined by basis vectors ***a***_1_–***a***_3_, is described by 4 σ parameters: σ_1–2_^x,y^, σ_3_^x,y^, σ_1–2_^z^ and σ_1–3_^z^. The first three describe the degree of deviation of the NP positions from the ideal ones in the direction parallel to the film substrate, while the fourth one describes the vertical deviation. The radii of the NPs are denoted by R_L_ and R_V_ for the directions parallel and perpendicular to the film substrate, respectively. The mean radius is denoted by *R*. The standard deviation of the size distribution is denoted by σ_R._ For more details about the paracrystal lattice and deviation parameters, as well as detailed GISAXS analysis, please see Reference [35]. The results of the numerical analysis are given in Table 2.

**Figure 2:**
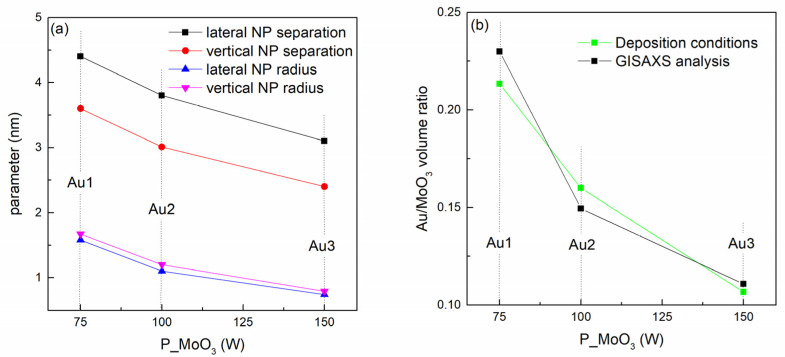
(**a**) Structural parameters of the films obtained by GISAXS analysis. (**b**) Ratio of the volumes of the Au nanoparticles and MoO_3_ matrix calculated from GISAXS parameters and from the deposition conditions (sputtering powers of Au and MoO_3_).

The structural properties of the NPs in the Au-based films, given in Table 2, are summarized in Figure 2. From Figure 2a it follows that all structural parameters including the inter-NP separation *a*, vertical separation *c* and the NP radii R_L_ and R_V_ all decrease with increasing MoO_3_ sputtering power, i.e., with decreasing Au percentage in the films. Figure 2b shows the comparison of the Au/MoO_3_ volume ratio obtained from the GISAXS analysis (parameters of the NP size and arrangement), and from the deposition conditions (sputtering powers of Au and MoO_3_). The good agreement between these two curves confirms the reliability of the GISAXS analysis.

The GISAXS analysis shows the decrease of all main NP structural parameters with the sputtering power of MoO_3_. The increase in the MoO_3_ sputtering power causes the increase in the number of its atoms on the growing surface during the deposition, while the number of the Au atoms is constant. The increased number of the matrix atoms obviously decreases the diffusion length of the Au atoms resulting in a smaller inter-nanoparticle distance *a* with increasing MoO_3_ power. The decrease in the diffusion length also results in the formation of smaller NPs, i.e., in the decrease of R_L_. The parameters describing the direction perpendicular to the film surface (parameters *c* and R_V_) also decrease with the MoO_3_ sputtering power. The reason is very probably the morphology of the growing surface that influences the self-assembly mechanism (see Reference [36]).

#### 3.1.2. Nanoparticle Internal Structure—Crystalline Properties

**Figure 3:**
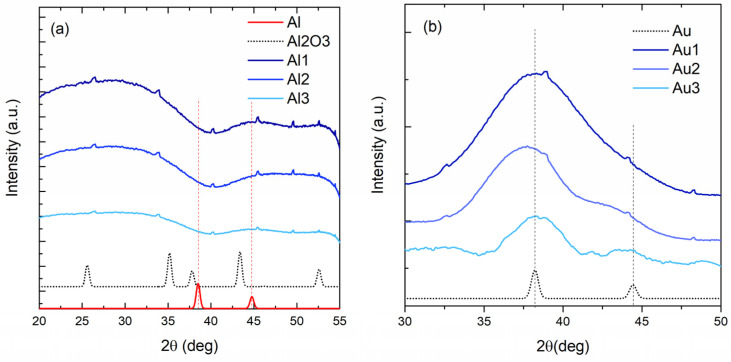
Grazing-incidence wide angle X ray scattering (GIWAXS) measurements of the (**a**) Al-based and (**b**) Au-based films. The tiny peaks with similar shapes, visible in most of the curves, are detector artefacts.

The crystalline structure of the films was explored by GIWAXS measurements. The curves measured on Al-based and Au-based films are shown in Figure 3a,b, respectively. The measurements support the GISAXS findings. In the curves of the Al-based films (Figure 3a) there are no Al-related crystalline peaks; however, broad Al_2_O_3_-related peaks are weakly observable. Al atoms are therefore very probably oxidized by the oxygen from the MoO_3_ matrix and residual gas from the deposition chamber, and there is no Al NP formation.

However, the Au-based films (Figure 3b) show clear Au-based (111) and (200) crystalline peaks at 38.1 and 44.3 degrees, respectively. The width of the peaks increases from Au1 to Au3 film, and the (200) peak becomes more visible. The width of the peak is related to the crystallite size; the wider peaks show smaller crystalline grains. Thus, the GIWAXS results show an increase in the Au crystallite size. The Au crystallite radii calculated from the width of the peaks are as follows: 0.7, 0.9 and 1.6 nm for the films Au1, Au2 and Au3, respectively. The obtained values are in good agreement with the Au NP size increase found by GISAXS (Table 2).

### 3.2. Optical Properties of the Films

**Figure 4:**
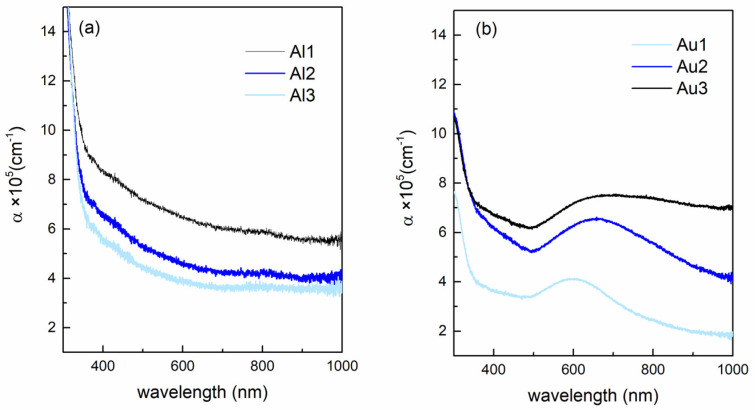
Optical properties of the films. Absorption coefficient vs. wavelength for (**a**) Al-based and (**b**) Au-based films.

The optical properties of the films (absorbance vs. wavelength and energy) are shown in Figure 4. The absorption coefficient of the Al-based films is shown in Figure 4a. Interestingly, the value of the absorbance decreases with an increasing amount of Al (Al1–Al3). The reason could be the formation of Al_2_O_3_ in the reaction of Al with O. The refractive index of alumina is lower than that of MoO_3_ in the measured range of wavelengths [37,38,39], so the increase in the amount of Al_2_O_3_ content in the film may reduce the total absorbance of the films, as observed experimentally. This is in agreement with the GIWAXS results that indicate formation of Al_2_O_3_ in the Al-based films.

The Au-based films show the opposite trend, so the overall absorption coefficient increases with increasing Au content (Au1–Au3) (Figure 4b). Additionally, the Au-based films show the presence of Au surface plasmons, with the maximum close to 580 nm for the film with the smallest Au NPs. The plasmon peak shifts toward higher wavelengths, and it becomes broader with increasing Au NP size. The broadening of the peak is the consequence of the broadening of the Au NP size distribution from A1 to A3 (see the parameter σ_R_, Table 2). Otherwise, the width of the plasmon peak should have the opposite behavior [31,40].

### 3.3. Electrical Properties of the Films

NPs embedded in oxide matrices are very interesting for nanotechnology applications due to their semiconductor nature, strong confinement effects and dependence of the material properties on the matrix in which the QDs are embedded. Therefore, knowledge of the electrical and transport properties of these materials is essential.

**Figure 5:**
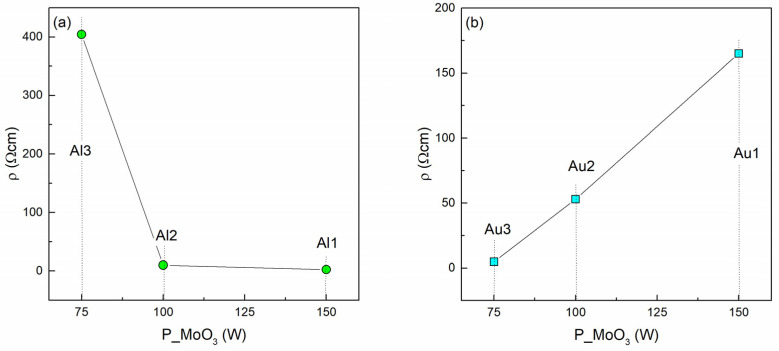
Resistivity as a function of sputtering power of (**a**) Al-based and (**b**) Au-based thin films.

The dependence of the measured resistivity of Al- and Au-based films on the MoO_3_ deposition power is given in Figure 5. The resistivity of pure MoO_3_ is about 10 times higher than the highest resistivity of the Al-based film (about 20 MΩcm [41]). It follows from Figure 5a that a decrease of the Al amount causes a drop of the material resistivity. The decrease of the resistivity with the Al content could have a similar origin to doping of some other oxide matrices with Al (like Al-doped ZnO-AZO for example [32]). In the case of AZO, an atomic substitution of Al to Zn in the ZnO crystal structure occurs, which provides a reduction in its electrical resistivity. We also observed a lack of Al nanoparticle formation and a drop in the overall absorption, which supports this assumption. However, a more detailed investigation is needed to explain in detail the effect of Al-doping of MoO_3_.

Interestingly, the Au-based films show the opposite trend (Figure 5b). The increase in the proportion of gold in the MoO_3_ matrix reduces the total resistivity of the films. In the Au-based films Au nanoparticles are formed, so another transport mechanism occurs.

**Figure 6:**
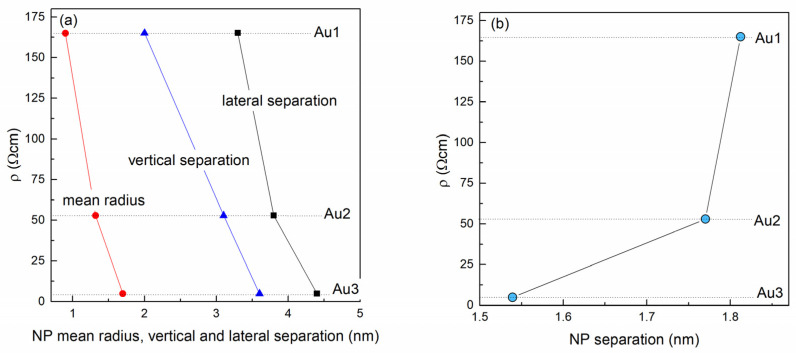
Resistivity as function of the (**a**) Au NP lattice parameters, (**b**) Au NP separation.

The dependence of the resistivity on the Au NP lattice parameters and Au NP separation are shown in Figure 6a,b, respectively. Figure 6a shows that the resistivity drops with all of the main structural parameters including the mean NP radius *R* and lateral and vertical NP lattice parameters (*a* and *c,* respectively). The more interesting parameter is the Au NP separation, which is calculated from the arrangement parameters of the Au nanoparticles and their radii. The dependence of the resistivity on the Au separation (closest distance to the neighbor) is shown in Figure 6b. From the figure it is evident that the resistivity significantly increases with the NP separation.

Several different transport mechanisms exist to describe the resistivity in quantum dot assemblies including, but not limited to, tunneling and hopping conduction [42]. Based on the results shown in Figure 6b, we believe that the main transport occurs by tunneling of charge carriers through MoO_3_ barriers between Au NPs [43,44]. In a simplified case, a rectangular potential barrier between NPs is assumed, where the probability of tunneling, and thus the conductivity, decreases exponentially with the nanoparticle distance. We intend to perform a more profound analysis of the transport mechanism on a larger series of samples in our future work.

## 4. Conclusions

We studied Au+MoO_3_ and Al+MoO_3_ thin films prepared by magnetron sputtering codeposition at room temperature. The structural parameters of these films and their main optical and transport properties were explored. We show that Al does not form nanoparticles in the MoO_3_ matrix, contrary to Au, which produces a 3D lattice of nanoparticles. The dependence of the parameters of the Au NP lattice on the deposition condition is shown. Tuning the deposition conditions, we can tune the size and separation of the Au NPs. The optical properties of the Al- and Au-based films also significantly differ. The Al-based films show a decrease of the absorption with increasing Al content. The Au-based films show the opposite trend, and in addition they show a size-dependent surface plasmon resonance peak. The Al- and Au-based films show opposite behavior of their electrical properties. While the resistivity for the Al-based films increases with the Al content, the resistivity decreases for the Au-based ones. We believe that Al incorporates in the MoO_3_ lattice, while the electrical transport in Au-based films occurs via tunneling of charge carriers between NPs. The studied material is very interesting for application in various nanotechnology devices.

## Figures and Tables

**Table 1:** Deposition parameters of the films. *P* indicates the sputtering power of the corresponding target, *d* is the film thickness determined from grazing-incidence small-angle X-ray scattering (GISAXS) measurements and Au/Al to MoO_3_ shows the targeted volume ratio of the metal to the matrix.

Sample/Par	*P*_Au/Al_ (W)	*P*_Au/Al_ (W/cm^2^)	P _MoO3_ (W)	P _MoO3_ (W/cm^2^)	*d* (nm)	Au/Al to MoO_3_ Volume Ratio
Au1	6	0.132	150	3.289	29.4	0.10
Au2	6	0.132	100	2.193	20.6	0.15
Au3	6	0.132	75	1.644	16.0	0.20
Al1	10	0.219	150	3.289	26.5	0.10
Al2	10	0.219	100	2.193	16.3	0.15
Al3	10	0.219	75	1.644	13.2	0.20

**Table 2:** Parameters of the Au QD lattices found by GISAXS analysis. *a* and *c* are the lateral and vertical separation of Au NPs, respectively; σ_1–3_^x,y,z^ are the deviation parameters, and R_L_ and R_V_ are the Au NP lateral and vertical radii respectively. All values are given in nm.

Sample/Par.	*a*	*c*	σ_1,2_^x,y^	σ_1,2_^z^	σ_3_^x,y^	σ_3_^z^	R_L_	R_V_	σ_R_
Au1	3.3	2.0	1.1	0.8	1.9	0.7	0.8	0.9	0.1
Au2	3.7	3.1	1.4	1.3	1.5	0.8	1.0	1.2	0.2
Au3	4.5	3.6	1.8	0.9	2.1	1.2	1.5	1.7	0.3

## Data Availability

Datasets available at: Mičetić, Maja (2021), “Structural, Optical and Electrical Properties of Al+MoO_3_ and Au+MoO_3_ Thin Films Prepared by Magnetron Codeposition”, Mendeley Data, V1, doi: 10.17632/8xdbb452nt.1
